# *Médecine Tropicale et Santé Internationale* s'engage contre la prédation éditoriale

**DOI:** 10.48327/mtsi.v3i4.2023.429

**Published:** 2023-12-08

**Authors:** Jean-Philippe CHIPPAUX, Jean-Paul BOUTIN, Michel DEVELOUX, Alain EPELBOIN, Pierre GAZIN, François MOUTOU, Jean-François PAYS, Eric PICHARD

**Affiliations:** SFMTSI. Hôpital Pitié-Salpêtrière – Pavillon Laveran, 47-83 Boulevard de l'Hôpital, 75013 Paris, France

**Keywords:** Revue prédatrice, Frais de publication, Libre accès diamant, Peer review, Creative Commons Attribution, PubMed^®^, Scopus^®^, Predatory journal, Publication fees, Diamond Open Access, Peer review, Creative Commons Attribution, PubMed^®^, Scopus^®^

## Abstract

La mise en garde contre les revues prédatrices s'intensifie. Destinées à capter les manuscrits avec la promesse d'une publication rapide, ces revues ont pour principal objectif de percevoir des frais de publication abusifs. Se prévalant parfois de facteurs d'impact imaginaires, elles ne sont pas indexées et n'apportent aucune garantie de visibilité, d'accessibilité et de pérennité de l'article publié. Et surtout, elles ne se préoccupent pas de la rigueur et de l'intégrité scientifique des travaux qu'elles éditent.

## Abstract (en)

Warnings against predatory journals get stronger. Designed to capture manuscripts with the promise of rapid publication, the main aim of these journals is to charge abusive publication fees. Sometimes boasting imaginary impact factors, they are not indexed and offer no guarantee of visibility, accessibility or durability of the published article. Above all, they have no concern for the rigor and scientific integrity of the work they publish.

La mise en garde contre les revues prédatrices s'intensifie. Destinées à capter les manuscrits avec la promesse d'une publication rapide, ces revues ont pour principal objectif de percevoir des frais de publication abusifs. Se prévalant parfois de facteurs d'impact imaginaires, elles ne sont pas indexées et n'apportent aucune garantie de visibilité, d'accessibilité et de pérennité de l'article publié. Et surtout, elles ne se préoccupent pas de la rigueur et de l'intégrité scientifique des travaux qu'elles éditent.

Plusieurs associations ou sociétés académiques se sont mobilisées pour faire connaître ce fléau. Des blogs privés veillent et renseignent. De nombreuses facultés ont créé des enseignements pour informer et prévenir leurs étudiants et jeunes chercheurs. Des éditeurs privés renommés abandonnent quelques-unes de leurs revues devenues déviantes. Certains chercheurs en sciences de la communication se sont essayés à établir des listes de revues prédatrices qui les ont attaqués pour diffamation.

Fin 2020, la Faculté de médecine de Sorbonne Université (France) a établi une liste des revues présumées « non-prédatrices » (https://sante.sorbonne-universite.fr/recherche/liste-des-revues-presumees-non-predatrices) qu'elle met périodiquement à jour. La première version, constituée entre octobre 2020 et mai 2021, est parue en août 2021; la dernière mise à jour date d'avril 2023. La Faculté invite ses chercheurs à soumettre leurs manuscrits aux revues de cette liste. Cette initiative de Sorbonne Université entend échapper au risque de poursuite judiciaire – puisqu'aucune revue prédatrice n'est citée – et recenser celles qui sont vertueuses. Cependant, la liste ne peut prétendre être exhaustive, ni constamment actualisée. Ainsi, le *Bulletin de la Société de Pathologie exotique* et *Médecine tropicale,* toutes deux arrêtées lors de la constitution de la liste, y figurent encore, tandis que *MTSI,* créée en janvier 2021, n'est toujours pas citée. Ceci doit nous inciter à préparer un argumentaire pour demander son insertion dans la liste. En outre, les revues en Libre accès diamant *(Diamond Open Access)* y sont minoritaires. Gratuites pour l'auteur comme pour le lecteur avec la totale liberté d'utilisation du contenu de l'article par tous, à condition de citer la source, elles se développent, particulièrement en Amérique hispanophone et lusophone, et constituent un modèle prometteur.

*MTSI,* organe scientifique de la Société francophone de médecine tropicale et santé internationale (SFMTSI), a fait suite au *Bulletin de la Société de pathologie exotique* au moment où cette société plus que centenaire entamait sa réforme. *MTSI* est l'héritière de toutes les revues françaises de médecine tropicale dont les publications se sont arrêtées avant 2020. *MTSI* lutte activement contre le recours aux revues prédatrices qui abusent auteurs comme lecteurs (parmi lesquels des décideurs). Les premiers, mal informés, pressés, bousculés par des échéances professionnelles ou académiques, attirés par la facilité comme par le marketing de ces éditeurs, espèrent une publication rapide de leurs travaux, sans toujours être conscients de leurs éventuelles insuffisances formelles ou conceptuelles qu'une revue scientifique aurait décelées. En outre, ils sont privés de leurs droits d'auteur – acquis par la revue prédatrice –, ce qui restreint l'accès à leurs données. De leur côté, les lecteurs accordent un crédit scientifique à des résultats non vérifiés et, de bonne foi, les diffusent dans leurs propres publications participant à la propagation de fausses informations.

La volonté profonde et revendiquée de la SFMTSI, comme du Comité de rédaction de *MTSI,* est d'offrir – dans tous les sens du terme – une tribune à l'expression scientifique dans son domaine de compétence. Elle met à la disposition des auteurs francophones une plateforme entièrement gratuite comprenant l'accompagnement à la bonne rédaction scientifique, l’évaluation par les pairs, la mise en page et la publication en ligne de leur manuscrit. Supprimant tous les coûts d'abonnement et d'achat en ligne, elle permet un accès direct et immédiat aux articles par les lecteurs. Elle garantit aux deux – auteur comme lecteur – la visibilité, grâce à l'indexation dans PubMed^®^, Scopus^®^, ainsi que la conservation et l'accès dans la durée aux textes archivés. *MTSI* offre la possibilité de publier à des auteurs francophones peu ou pas familiarisés avec la langue anglaise, leur évitant des frais de traduction, et facilite par là même l'accès à des résultats qui resteraient méconnus autrement.

En choisissant que ses articles soient en Libre accès diamant^1^, *MTSI* participe au mouvement de la Science ouverte qui vise à rendre accessibles à tous, les données produites par la recherche scientifique, particulièrement dans l'espace francophone.

